# Carotid atherosclerotic plaques standardized uptake values: methodological issues on reproducibility and accuracy

**DOI:** 10.1186/s13550-017-0308-x

**Published:** 2017-09-07

**Authors:** Siamak Sabour

**Affiliations:** 1 Safety Promotion and Injury Prevention Research Center, Shahid Beheshti University of Medical Sciences, Tehran, IR Iran; 2 Department of Clinical Epidemiology, Shahid Beheshti University of Medical Sciences, Tehran, IR Iran

**Keywords:** ^18^F–FDG, Carotid atherosclerotic plaque, Positron emission tomography-computed tomography, Reproducibility, Accuracy

## Correspondence/Findings

To assess accuracy and precision of the positron emission tomography-computed tomography (PET-CT) carotid standardized uptake values (SUV) of 18F–fluorodeoxyglucose (18FDG) as an inflammatory biomarker for determining cerebrovascular diseases such as stroke, methodology and statistical issues should be taken into account. Otherwise, misleading messages will be the main outcome of such research. Briefly, confusing accuracy and precision will mainly produce misleading messages.

I was interested to read the paper by Giannotti N and colleagues published in the Dec 2017 issue of EJNMMI Res [1]. Positron emission tomography-computed tomography (PET-CT) carotid standardized uptake values (SUV) of ^18^F–fluorodeoxyglucose (^18^FDG) have been proposed as an inflammatory biomarker for determining cerebrovascular diseases such as stroke. Consideration of varying methodological approaches and software packages is critical to the calculation of accurate SUVs in cross-sectional and longitudinal patient studies. They aimed to investigate whether or not carotid atherosclerotic plaque SUVs are consistent and reproducible between software packages [1]. ^18^FDG-PET SUVs of carotids were taken in 101 patients using two different software packages [1]. Data from five to seven anatomical sites were measured. A total of ten regions of interest (ROI) were drawn on each site. Based on their results statistically significant differences in SUV measurements, between the two software packages, ranging from 9 to 21.8% were found depending on ROI location. In 79% (*n* = 23) of the ROI locations, the differences between the SUV measurements from each software package were found to be statistically significant. They highlighted the importance of standardizing all aspects of methodological approaches to ensure accuracy and reproducibility.

However, reproducibility (precision, repeatability, reliability, or interchangeability) and accuracy (validity) are two completely different methodological issues [2–8]. The methodological approach and statistical estimates to assess these issues are completely different. For reliability purposes, our approach should be individual based. It means for continues variables, intraclass correlation coefficient (ICCC) absolute agreement single measure should be considered. 9 to 21% statistically significant differences in SUV measurements between the two software packages indicate that the authors did not applied this approach. They considered global average approach for reliability which is a common mistake and usually applied to assess accuracy of a test compared to a gold standard. It is crucial to know that a test can be accurate with no reliability and vice versa. Moreover, statistically significant should not be considered in reproducibility analysis because it dramatically depends on the sample size [2–8]. Finally, confusing precision and accuracy will mainly produce misleading messages.
